# Lobular endocervical glandular hyperplasia: Two case report and literature review

**DOI:** 10.1097/MD.0000000000040968

**Published:** 2024-12-27

**Authors:** Shenglan Tang, Xuelu Jiang, Xue Wang

**Affiliations:** a Department of the First School of Clinical Medicine, Zhejiang Chinese Medical University, Hangzhou, Zhejiang Province, People’s Republic of China; b The First Affiliated Hospital of Zhejiang Chinese Medical University (Zhejiang Provincial Hospital of Chinese Medicine), Hangzhou, Zhejiang Province, People’s Republic of China.

**Keywords:** diagnosis, gastric-type adenocarcinoma, lobular endocervical glandular hyperplasia, pathology, therapy

## Abstract

**Rationale:**

Lobular endocervical glandular hyperplasia is a rare proliferative lesion of the endocervical glands with an unclear pathogenesis. The condition lacks specific clinical manifestations and signs and often leading to clinical underdiagnosis and misdiagnosis.

**Patient concerns:**

A 27-year-old female patient presented with a history of abnormal vaginal bleeding over the previous 3 months. An ultrasound examination revealed the presence of a cystic solid structure in the posterior wall of the cervix.

The second patient is a 25-year-old woman who has undergone 3 cervical cystectomies over the past 9 years due to recurrent cervical cysts. It is regrettable to report that the cyst has now recurred.

**Diagnoses:**

Lobular endocervical glandular hyperplasia.

**Interventions:**

In consideration of the fertility needs of both patients, the gynecologists conducted a comprehensive imaging evaluation and detailed preoperative discussion, and performed ultrasound-guided hysteroscopic excision of cervical lesions and transvaginal excision of cervical lesions.

**Outcomes:**

Following treatment, the 2 patients exhibited a complete resolution of their clinical symptoms. Subsequent follow-up examinations demonstrated that both patients were exhibiting favorable outcomes.

**Lessons:**

The objective of this study is to present 2 cases of lobular endocervical glandular hyperplasia in order to facilitate a greater understanding of this rare disease among gynecologists. Furthermore, this study will describe the potential use of ultrasound-guided hysteroscopic excision of cervical lesions and transvaginal hysteroscopic excision of cervical lesions as a treatment modality for lobular endocervical glandular hyperplasia patients with fertility needs.

## 1. Introduction

Cervical cancer is the fourth most common cancer type among women globally. The International endocervical adenocarcinoma criteria and classification classifies cervical adenocarcinoma into 2 distinct types: human papilloma virus (HPV)-associated and non-HPV-associated.^[1]^ The former is more prevalent than the latter. As HPV vaccines and cervical cancer screening become more widely available, the incidence of HPV-associated cervical cancer will decrease, while the relative incidence of non-HPV-associated cervical cancer will increase. Amongst non-HPV-associated cervical adenocarcinoma subtypes, the gastric-type endocervical adenocarcinoma (GAS) is of particular concern due to its highly malignant nature, poor prognosis, and tendency for misdiagnosis.^[2]^ Lobular endocervical glandular hyperplasia (LEGH) is a rare proliferative lesion of the endocervical glands with an unclear pathogenesis, insidious onset, atypical symptoms, and diagnostic difficulties. LEGH is closely related to GAS, with atypical LEGH being regarded as a precancerous lesion of GAS. This report discusses 2 LEGH cases and the diagnostic and treatment experiences.

## 2. Case presentation

The first patient was a 27-year-old woman with a history of a cystic solid mass on the posterior wall of the cervix, which was discovered during a physical examination >10 months ago. However, this finding was not given the appropriate level of attention. More than 3 months prior, the patient had developed abnormal vaginal bleeding, which was not accompanied by any discernible etiology and was devoid of abdominal pain or other discomfort. The results of cervical cancer screening via a regular annual examination were normal, and the patient was unvaccinated against HPV. The patient had a history of sexual intercourse and was unmarried and childless. A review of the family history revealed no evidence of neoplasia or genetic disorders.

Following admission, a gynecological examination was conducted. A few brown vaginal discharge was observed, and the surface of the cervix was found to be smooth. The size of the cervical mass was approximately 5.0*4.0 cm, with a hard texture. The parietal uterus was not involved.

**Figure 1.**
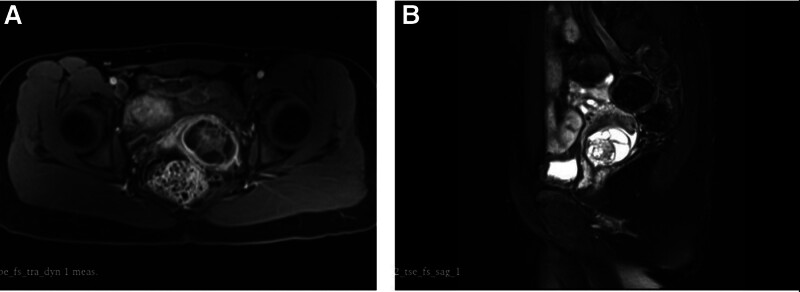
MRI images of patient 1. MRI = magnetic resonance imaging.

Ultrasonography revealed the presence of a cystic solid mass in the posterior wall of the cervix, situated in close proximity to the external orifice. The mass exhibited a size of approximately 3.8*3.6*4.3 cm, maintaining a regular morphology and clear boundary. Within the mass, a septated and solid echogenic mass could be identified, accompanied by a cystic portion that appeared as a fine light spot. The envelope and solid portion of the blood flow signal exhibited greater richness, with an resistance index of 0.67. Pelvic magnetic resonance imaging (MRI) revealed that the posterior part of the cervix exhibited a round-like cystic lesion with an envelope and septation. Septation, T1-weighted imaging, and T2-weighted imaging demonstrated a mixed high signal, while the solid component of the diffusion weighted imaging showed a slightly high signal (Fig. 1). The envelope and septation exhibited obvious enhancement following enhanced scanning, and the size and shape were within normal limits. The pelvic wall structure was observed to be normal, and no enlarged lymph nodes were identified within the pelvis.

**Figure 2.**
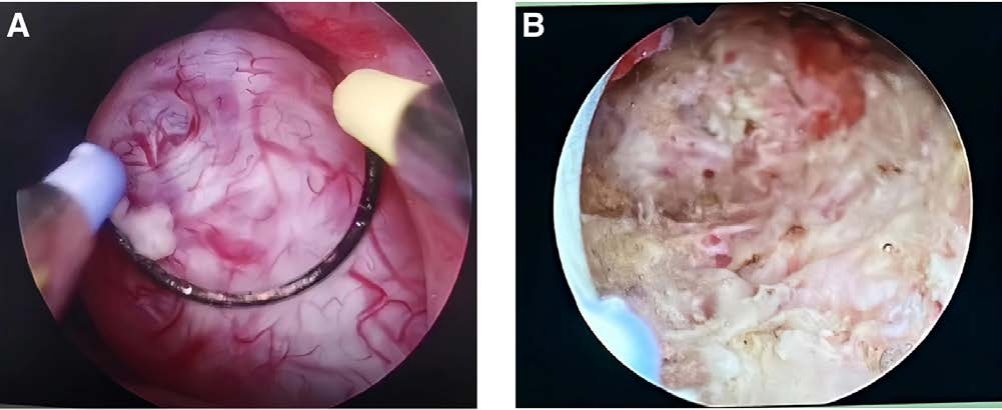
Hysteroscopic photographs of patient 1. (A) Depicts the intrauterine redundant organisms observed via hysteroscopy, while (B) illustrates the hysteroscopic image subsequent to the removal of the cervical cyst.

**Figure 3.**
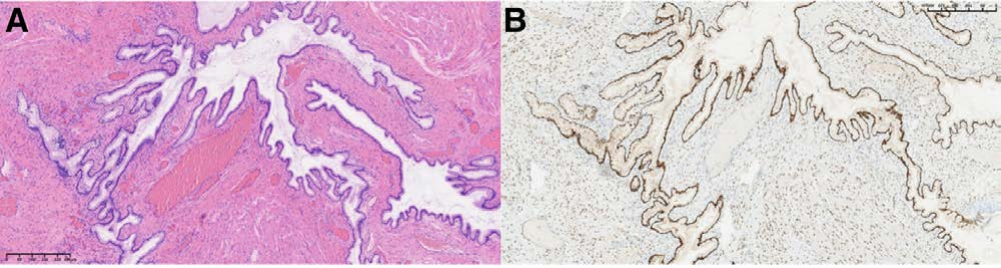
Pathological findings of patient 1.

In light of the patient’s condition and the practitioner’s previous experience, a hysteroscopic cervical lesion resection and a transvaginal cervical lesion resection were performed under ultrasound guidance (Fig. 2). Following intravenous general anesthesia, the cervix was exposed, revealing a leiomyomatous organism measuring approximately 4.5*4.0 cm on the posterior lip of the cervix. The organism exhibited a cystic surface, abundant blood vessels, and poor exposure of the cervix. The cervix was excised with an electrosurgical knife, and hemostasis was achieved through surface electrocoagulation. Following this, the bleeding was stopped after suturing with a No. 1 absorbable suture at 9 o’clock. A hysteroscopic examination revealed that the cervical canal was cylindrical in shape, and the root of the cervical polyp was visible at the external cervical opening. The uterine cavity was observed to be inverted pear-shaped, with the presence of multiple polypoid formations in the middle and lower uterus. The largest of these was approximately 0.8*0.5 cm in size, exhibiting uneven endometrial hyperplasia. Bilateral tubal openings were also visible, while the uterine fundus and horns appeared to be in a normal state. The bipolar diathermy ring was employed to halt the hemorrhage at the root of the cervical polyps, and the root of the uterine polyps was excised to remove the polyps. Following intravenous general anesthesia, the cervix was exposed, revealing a leiomyomatous organism measuring approximately 4.5*4.0 cm on the posterior lip of the cervix. The organism exhibited a cystic surface, abundant blood vessels, and poor exposure of the cervix. The cervix was excised with an electrosurgical knife, and hemostasis was achieved through surface electrocoagulation. Following this, the bleeding was stopped after suturing with a No. 1 absorbable suture at 9 o’clock. A hysteroscopic examination revealed that the cervical canal was cylindrical in shape, and the root of the cervical polyp was visible at the external cervical opening. The uterine cavity was observed to be inverted pear-shaped, with the presence of multiple polypoid formations in the middle and lower uterus. The largest of these was approximately 0.8*0.5 cm in size, exhibiting uneven endometrial hyperplasia. Bilateral tubal openings were also visible, while the uterine fundus and horns appeared to be in a normal state. The bipolar diathermy ring was employed to halt the hemorrhage at the root of the cervical polyps, and the root of the uterine polyps was excised to remove the polyps. The postoperative pathological report indicated LEGH. Immunohistochemical staining results: ER (+), PR (+), MUC (+), MUC6 (+), p16 (focally +), and Ki-67 (5% +). The 27-year-old patient, who was unmarried and childless, declined a total hysterectomy (Fig. 3). We therefore recommended a complete gastroenterological examination and subsequent close follow-up after surgery.

Another patient was a 25-year-old woman who was admitted to a local hospital in 2015 due to a self-conscious vaginal mass detachment. A diagnosis of a cervical cyst was rendered, and the patient subsequently underwent hysteroscopic excision of the cyst. The second cervical cystectomy was performed in 2019 due to the recurrence of the condition. In 2022, a recurrence occurred, necessitating the collection of a sample for biopsy of the cervical mass. The subsequent pathological examination revealed the presence of chronic inflammation of the uterine mucosa with adenocyst formation. Given the relatively limited size of the mass, a decision was made to postpone treatment for the time being. A subsequent MRI scan conducted in 2023 revealed a markedly enlarged honeycomb-like cervical mass. The patient was subsequently subjected to a second cervical cystectomy. The postoperative pathology report indicated the presence of chronic inflammation of the uterine cervix mucosa, accompanied by the formation of multiple glandular cysts and glandular hyperplasia. Furthermore, periglandular smooth muscle tissue demonstrated tumor-like hyperplasia. It is regrettable to report that a third relapse occurred in 2024. The results of cervical cancer screening via a regular annual examination were normal, and the patient was unvaccinated against HPV. The patient had a history of sexual intercourse and was unmarried and childless. A review of the family history revealed no evidence of neoplasia or genetic disorders.

Upon admission, a gynecological examination was conducted, revealing the presence of some transparent vaginal discharge. The cervix of the uterus was ill-defined and predominantly covered by a mass, and a cystic solid mass measuring approximately 11.0*8.0 cm was identified in the supracervix. This mass was softer in texture and exhibited no parietal uterine involvement.

**Figure 4.**
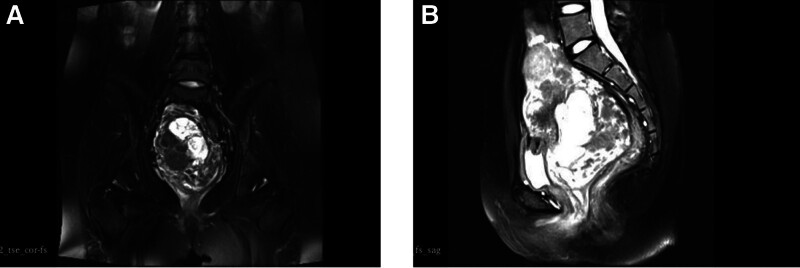
MRI images of patient 2. MRI = magnetic resonance imaging.

dUltrasonography indicates that the right wall and posterior wall of the cervical canal are markedly thickened and structurally disorganized. A solid, predominantly lobulated, hypoechoic mass is observed, exhibiting an irregular morphology and measuring approximately 11.1*7.7*6.8 cm. Its internal echogenicity is uneven, it lacks an obvious envelope, and its blood flow signal is slightly elevated. The mucosal layer of the cervix was markedly thickened, and multiple cystic cavities of varying sizes were observed. Additionally, several cystic predominantly honeycomb nodules were present within the cervical canal, with the largest measuring approximately 3.5*2.8 cm. These nodules exhibited an oval morphology, distinct borders, and multilocular septations. However, no discernible blood flow signals were evident. MRI of the pelvis indicated the presence of a multicompartmental cystic signal shadow in the cervix. The signal exhibited mixed isohigh signals in T2-weighted imaging, low signals with some high signals in T1-weighted imaging, high signals in diffusion weighted imaging, and low signals in apparent diffusion coefficient. No enlargement of lymph nodes was observed in the pelvic region (Fig. 4).

**Figure 5.**
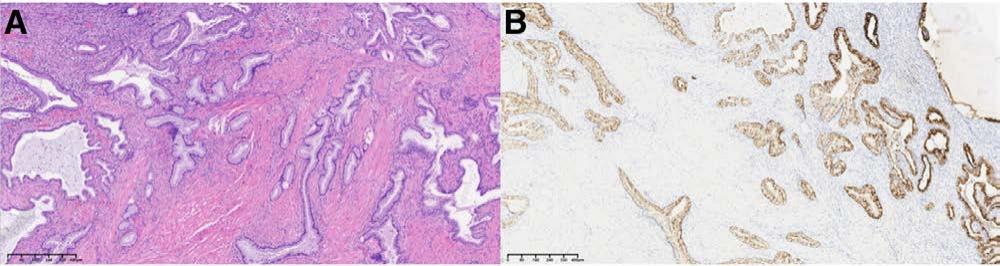
Pathological findings of patient 2.

Given the patient’s age (25 years), marital status, and childlessness, as well as her stated reproductive plan and the absence of malignant pathology in the pathology reports from 3 previous surgeries, a recommendation was made for conservative surgical treatment. As a result of the patient’s large cervical mass, a cervical conical resection could not be carried out. Instead, ultrasound-monitored hysteroscopic cervical mass removal and transvaginal cervical mass removal were conducted. Following intravenous general anesthesia, the cervix was exposed, and a large mass was observed spanning points 1 to 11, with an approximate size of 6 by 8 centimeters. A TV hysteroscope was introduced under ultrasound guidance into the cervical canal, where it was observed that the canal wall was honeycombed with multiple cystic cavities. Additionally, mucus-like secretions were noted to be present within the cystic cavities. The larger cysts in the cervical canal had a size of approximately 6 by 5 centimeters and were located in the mid-cervical segment, visible in the internal cervical os. The cyst was successfully removed by the bipolar electrosurgical ring, with a complete removal of the cysts. The cyst was extracted from the cervical canal, concluding the hysteroscopic procedure. The cysts were removed from the anterior and posterior walls of the cervix via vaginal approach under ultrasound guidance, and the cervical wound was closed with 2-0 absorbable suture, thereby restoring the normal morphology of the cervix. The pathological findings of cervical are LEGH with gastric (pyloric) metaplasia, which is combined with neoplastic hyperplasia of the smooth muscle. The pathology of the cervical canal demonstrated the presence of LEGH with gastric (pyloric) hyperplasia. The immunohistochemical staining of smooth muscle revealed the presence of Ki-67(<2%+), SMA(+), Desmin(+), HMB-45(−), S-100(−), MelanA(−), TTF-1(−), the immunohistochemistry of LEGH demonstrated Ki-67 (<1%+), MUC5AC(focal +), and MUC-6(+) (Fig. 5). In consideration of the pathological findings and the specific condition of the patients, we advised them to maintain close follow-up after surgery, pursue childbearing as soon as medically feasible, and consider undergoing hysterectomy should circumstances warrant. Both patients have now undergone the most recent follow-up examination and are exhibiting satisfactory outcomes.

## 3. Discussion

LEGH is a rare proliferative lesion of the endocervical glands with an unclear pathogenesis. LEGH was initially proposed by Nucci et al^[3]^ in 1999. As the comprehension of cervical adenoepitheliopathy advanced and representative cases were accumulated, researchers discovered that LEGH exhibited analogous clinicopathologic characteristics and immunephenotypes as GAS, suggesting a potential close association between the two.

The majority of patients diagnosed with LEGH are premenopausal women. The clinical manifestations of this condition include vaginal discharge or mass, or asymptomatic incidental findings. Computed tomography or MRI reveals the presence of polycystic lesions, exhibiting a distinctive floral arrangement of small cysts, which collectively form a “Cosmos bipinnatus pattern.”^[4]^ LEGH is primarily diagnosed through a pathological diagnosis. Macroscopically, the surface of the incision displays cystic spaces, which are typically situated in the upper portion of the cervix, in close proximity to the internal orifice.^[5]^ Microscopically, the cervical glandular tissue displays small glands arranged in lobular aggregates around a central macroglandular gland. The glands are lined with mucous epithelium, pale eosinophilic cytoplasm, and small nuclei confined to the basal part of the body exhibit a mild morphology. Additionally, occasional intestinal epithelial hyperplasia is present, particularly within the central gland. Larger cysts may extend deeper into the wall of the cervix, and localized glands may display a diffuse pattern rather than the typical lobular structure. Immunohistochemical analysis reveals the following: (1) The component glandular cells demonstrate cytoplasmic immunoreactivity for MUC6 and/or HIK1083; (2) the nuclei exhibit minimal to no reactivity for estrogen and progesterone receptors (ER, PR); (3) cytokeratin 7 (CK7) is positive and cytokeratin 20 (CK20) is negative; (4) CEA is positive only in the apical part of columnar cells; (5) p16 is negative or strongly weakly negative; (6) CEA is positive only in the apical part of columnar cells; and (7) The Ki-67 index is very low, with little to no positivity visible and only occasional scattered positivity. Cytologic features manifest as clusters of yellow-orange or golden-yellow glandular cells with intracytoplasmic mucin on smear preparations.^[6,7]^ In the event of heterogeneity in the glandular structure of LEGH and its overlying epithelium, the condition is referred to as atypical lobular endocervical glandular hyperplasia (ALEGH), which is regarded as a precancerous lesion of GAS/minimal deviation adenocarcinoma of the uterine cervix. In addition to the pathological features of LEGH, ALEGH is accompanied by at least 4 atypical morphologies, including enlarged nuclei, irregular nuclear membranes, conspicuous nucleoli, coarse chromatin, loss of polarity, occasional nuclear fission, apoptotic vesicles or nuclear debris in the lumen of the ducts, and papillae or epithelial folds accompanied by a ciliated fiber-vascular stroma.^[7]^

The insidious onset of LEGH, atypical symptoms, and the typical absence of a positive screening result for HPV make early screening and diagnosis of LEGH challenging. Clinically, it is important to differentiate LEGH from GAS and Peutz-Jeghers syndrome (PJS).

GAS is a non-HPV-associated cervical adenocarcinoma that exhibits gastric differentiation. The clinical symptoms and morphology of GAS are analogous to those of LEGH, frequently manifesting as vaginal mucus-like or watery discharge, pelvic and abdominal masses, and so forth. The cervix may be enlarged and smooth, appearing as a “barrel-shaped” cervix or as an ectopic mass, which may be cauliflower-like, hard, nodular, or other forms. However, the mass of GAS is not clearly demarcated and may involve a number of different structures, including the uterine body, the entire periphery of the cervical canal, the deep myometrial layer, and even the plasma membrane layer. Additionally, it may extend to the parietal uterus, as well as lymphovascular space invasion and perineural invasion, among other potential locations. Morphologic features include the presence of large amounts of clear, foamy, or pale eosinophilic cytoplasm with well-defined cell borders, as well as a generally low karyoplasma ratio and irregular distribution of nuclei at the base of the glands.^[8]^ The immunohistochemical manifestations of gastric-type mucus are characterized by the presence of MUC6 and HIK1083. (1) The combined detection of these markers can be utilized for the diagnosis of GAS and its spectrum of lesions; (2) p16 is typically negative or focally positive in GAS, and approximately 8.5% of patients may exhibit strong diffuse positivity for p16; (3) p53 mutant expression is observed in approximately 50% of patients; (4) estrogen and progesterone receptors (ER, PR) are predominantly negative; (5) CK7 and CK20 are mostly focally positive, or diffusely positive; (6) the Ki67 proliferation index is low, typically < 40%; and (7) Periodic Acid-Schiff staining reveals the presence of powder-stained mucus, which also offers a certain degree of auxiliary diagnostic value.^[8,9]^ The immunophenotype of GAS is complex and variable, necessitating a combination of morphological characteristics for a definitive pathological diagnosis.

LEGH can also manifest in patients with PJS who have LKB1/STK11 mutations. PJS can result in a range of disorders, including gastrointestinal misshapen polyps, mucosal pigmented patches of the skin, gastric adenocarcinoma, mucinous tumors of the ovaries, and sex cord mesenchymal tumors with annular tubules. The prevalence of GAS in PJS is approximately 15% to 30%. Consequently, endoscopy and genetic counseling are recommended if LEGH is detected.^[10]^

The simultaneous presence of mucinous lesions in 2 or more sites of the female genital tract is referred to as synchronous mucinous metaplasia and neoplasia of the female genital tract. This condition is characterized by gastric differentiation. Consequently, the detection of LEGH/ALEGH should prompt the consideration of synchronous mucinous metaplasia and neoplasia of the female genital tract, with a focus on investigating other potential sites, including further pelvic and abdominal imaging, and diagnostic curettage when appropriate.^[11,12]^

The 2 LEGH cases reported in our study were aged 25 and 27 years, which is below the age range typically considered high-risk for LEGH. The clinical symptoms were characterized by the prolapse of a vaginal mass and abnormal vaginal bleeding, respectively, and were not specific. It is therefore important to differentiate LEGH from other diseases that may present with vaginal bleeding, including cervical cancer, endometriosis, endocrine disorders, and HPV-associated cervical cancer. A gynecological examination is of great importance in the diagnosis of LEGH. In both cases, we observed increased cervical secretions and visible masses. Imaging revealed the presence of a well-defined cystic solid mass in both cases. Furthermore, cytologic screening and HPV testing yielded negative results. The gastrointestinal findings in both patients were unremarkable.

At present, there is no consensus regarding the standardized criteria for LEGH treatment in clinical practice. This presents a significant challenge in selecting an appropriate treatment option. The majority of scholars concur that hysterectomy represents a reasonable treatment option. It is imperative that pathologists examine the tissue in multiple sections to rule out the possibility of coexisting invasive cancers and to prevent misdiagnosis. Patients diagnosed with LEGH and ALEGH who undergo hysterectomy have a 100% survival rate at 5 years. Cervical conization is regarded as an unsuitable procedure due to the lesion’s location and the typical positive surgical margins. Cervical conization is only indicated for patients who have fertility requirements or who do not wish to undergo hysterectomy. It is recommended that total hysterectomy be performed following delivery or that active surveillance be employed for patients in whom ALEGH is not observed or in whom lesions are more severe and have negative surgical margins. In the present case, both patients were unmarried and infertile, with a desire for fertility and the presence of large masses. In particular, the second patient presented with multiple recurrent LEGH, which presented a challenge in terms of attempting to remove as much of the lesion as possible while preserving their fertility. Ultimately, the medical team opted for a hysteroscopic cervical lumpectomy and a transvaginal cervical lumpectomy to remove the cervical cysts, followed by suturing of the cervix to restore its normal shape. Furthermore, it was advised that both patients undergo a hysterectomy in the event of disease recurrence following delivery. The preliminary outcomes of the procedure were favorable, however, further long-term monitoring of the patients is required. Some studies have indicated that the presence of certain characteristics on MRI scans, such as enlarged cystic lesions, solid areas, or infiltrative borders, and cervical cytology findings suggestive of an increased degree of atypical adenocytosis, may indicate malignant transformation of cervical polycystic lesions.^[13]^ These findings may inform the postoperative management plan for our 2 patients.

## 4. Conclusion

LEGH is a rare endocervical glandular hyperplasia with an uncertain etiology. It has been demonstrated that LEGH exhibits analogous clinicopathological characteristics and immunophenotyping to those observed in GAS, thereby indicating the potential for a close correlation between the two. It is therefore imperative that clinicians implement enhanced management strategies for patients presenting with cervical polycystic lesions. It is recommended that the possibility of precancerous cervical lesions be considered in patients presenting with large cervical cysts and increased vaginal discharge or vaginal bleeding, even in the absence of positive results on cervical cytology and HPV testing. This is particularly crucial in patients with distinctive imaging manifestations who require heightened vigilance for LEGH. Ultrasound-guided hysteroscopic cervical tumor excision and transvaginal cervical tumor excision are viable treatment options for patients with LEGH who have fertility requirements, but close long-term follow-up is still essential after surgery.

## Author contributions

**Conceptualization:** Shenglan Tang, Xuelu Jiang, Xue Wang.

**Data curation:** Shenglan Tang, Xue Wang.

**Formal analysis:** Xuelu Jiang, Xue Wang.

**Writing – original draft:** Shenglan Tang.

**Writing – review & editing:** Shenglan Tang, Xuelu Jiang, Xue Wang.
